# Efficient network immunization under limited knowledge

**DOI:** 10.1093/nsr/nwaa229

**Published:** 2020-09-03

**Authors:** Yangyang Liu, Hillel Sanhedrai, GaoGao Dong, Louis M Shekhtman, Fan Wang, Sergey V Buldyrev, Shlomo Havlin

**Affiliations:** Department of Systems Science, National University of Defense Technology, Changsha 410073, China; Department of Physics, Bar-Ilan University, Ramat Gan 5290002, Israel; Faculty of Science, Jiangsu University, Zhenjiang 212013, China; Department of Physics, Bar-Ilan University, Ramat Gan 5290002, Israel; Network Science Institute, Northeastern University, Boston, MA 02115, USA; Department of Physics, Bar-Ilan University, Ramat Gan 5290002, Israel; Department of Physics, Yeshiva University, New York, NY 10033, USA; Department of Physics, Bar-Ilan University, Ramat Gan 5290002, Israel

**Keywords:** percolation, complex networks, network immunization, critical phenomena

## Abstract

Targeted immunization of centralized nodes in large-scale networks has attracted significant attention. However, in real-world scenarios, knowledge and observations of the network may be limited, thereby precluding a full assessment of the optimal nodes to immunize (or quarantine) in order to avoid epidemic spreading such as that of the current coronavirus disease (COVID-19) epidemic. Here, we study a novel immunization strategy where only *n* nodes are observed at a time and the most central among these *n* nodes is immunized. This process can globally immunize a network. We find that even for small *n* (≈10) there is significant improvement in the immunization (quarantine), which is very close to the levels of immunization with full knowledge. We develop an analytical framework for our method and determine the critical percolation threshold *p*_*c*_ and the size of the giant component *P*_∞_ for networks with arbitrary degree distributions *P*(*k*). In the limit of *n* → ∞ we recover prior work on targeted immunization, whereas for *n* = 1 we recover the known case of random immunization. Between these two extremes, we observe that, as *n* increases, *p*_*c*_ increases quickly towards its optimal value under targeted immunization with complete information. In particular, we find a new general scaling relationship between |*p*_*c*_(∞) − *p*_*c*_(*n*)| and *n* as |*p*_*c*_(∞) − *p*_*c*_(*n*)| ∼ *n*^−1^exp(−α*n*). For scale-free (SF) networks, where *P*(*k*) ∼ *k*^−γ^, 2 < γ < 3, we find that *p*_*c*_ has a transition from zero to nonzero when *n* increases from *n* = 1 to *O*(log *N*) (where *N* is the size of the network). Thus, for SF networks, having knowledge of  ≈log *N* nodes and immunizing the most optimal among them can dramatically reduce epidemic spreading. We also demonstrate our limited knowledge immunization strategy on several real-world networks and confirm that in these real networks, *p*_*c*_ increases significantly even for small *n*.

## Teaser

A novel network immunization strategy is proposed and studied to provide a theoretical framework for understanding and carrying out efficient immunization with limited knowledge.

## INTRODUCTION

Networks play a crucial role in many diverse systems [1–11]. Connectivity of components is critical for maintaining the functioning of infrastructures like the Internet [12] and transportation networks [13], as well as for developing efficient immunization against epidemics [14] and the spread of information in social systems [15]. Because of this importance, researchers have long focused on how a network can be optimally immunized or fragmented to prevent epidemics or maintain infrastructure resilience [16–20]. Many approaches have used percolation theory from statistical physics to prevent the spread of viruses or assess network resilience under the infection or failure of some fraction of nodes or links [21–32].

Early studies in networks found that immunizing real networks against an epidemic is highly challenging due to the existence of hubs that prevent eradication of the virus even if many nodes are immunized [33–35]. These largest degree nodes, are targeted, the network can quickly be separated, leading to immunity or failure [36]. However, previous models of targeted immunization have assumed full knowledge of the network structure that in most cases is not available. Research has shown that even those in control of a network often have knowledge of only a small portion of the whole network structure [37–41]. This has been further demonstrated with the current coronavirus disease 2019 (COVID-19) epidemic where the detailed social network of individuals is unknown.

In this paper, we study targeted immunization in networks with limited knowledge. Note that the methodology equally applies to efficient attacks (where the targeted nodes are removed) on networks. We assume that at each stage, *n* nodes are observed and the node with highest degree is immunized and thus unable to continue spreading infection. This procedure is repeated until a 1 − *p* fraction of nodes are immunized. In particular, our model could apply to a situation where several cooperative teams are sent to immunize a network and each team has access to information on a small subset (*n* nodes) of the network. We develop a theoretical framework for this model of immunization with limited information using percolation theory for networks with arbitrary degree distribution. In the limit of *n* = *N* we recover prior work on targeted immunization [36], whereas for *n* = 1 we recover the case of random immunization [34,35]. We observe excellent agreement between our theoretical framework and simulation results regarding the efficiency of the immunization as measured by both the critical threshold *p*_*c*_ and the size of the giant component *P*_∞_ for *p* > *p*_*c*_ as a function of *n*. We note that a smaller giant component implies improved immunization, whereas a larger critical threshold *p*_*c*_ is also more efficient since fewer quarantines/vaccines are needed to stop the epidemic. We find a scaling relationship between *n* and *p*_*c*_ for both Erdős-Rényi (ER) and scale-free (SF) networks, which we determine both analytically and through simulations. Surprisingly, we find that *p*_*c*_ quickly reaches a large value and a plateau even for relatively small *n* (of order 10), after which increasing *n* has negligible effect on *p*_*c*_. This suggests that obtaining information on even a small number (*n*) of people can lead to significant mitigation of the epidemic.

## RESULTS

### Model

Let *G*(*V*, *E*) be a network where *V* and *E* are the sets of nodes and edges, respectively. The number of nodes in the network is *N* = |*V*|. We assume that an immunizing team has limited knowledge of the overall network structure and instead possesses only limited information on several nodes. Specifically, we randomly select *n* nodes for which the immunizing team is assumed to obtain information on their node degree. We then immunize the node with the highest degree among these *n*. This could be performed by many teams such that they collectively immunize a fraction of 1 − *p* nodes. The process leads to identical results if instead of immunizing the nodes they are put into quarantine. We note that the immunizing team only retains knowledge of the initial node degree and does not reduce the degree based on any neighbors that might have been immunized or quarantined. This is due to the fact that when asking individuals about their neighbors, the individuals are unlikely to know that a neighbor has recently been immunized or quarantined.

**Figure 1.**
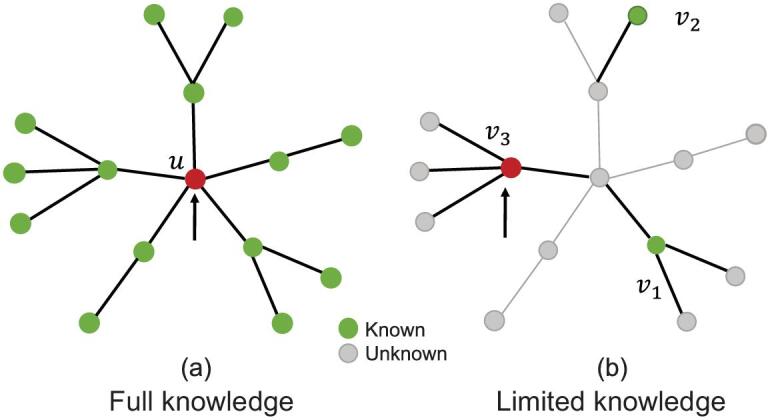
Schematic illustration of our limited knowledge immunization strategy. The immunizer is able to observe the degree of nodes that are colored green, while the gray nodes are unknown. (a) For the classical targeted immunization, one has complete information on the global structure of the network and chooses the highest degree node (*u*) to immunize. (b) Here the case of an individual with limited knowledge of the network is demonstrated. In this figure, we set *n* = 3 and only the degrees of nodes $v$_1_, $v$_2_ and $v$_3_ are known. Given this limited information, the immunizer would choose to immunize $v$_3_, being unaware that an unobserved higher degree node exists. At the next immunization, only nodes that have not been immunized yet will be observed.

In Fig. 1, the limited information immunization is illustrated and compared to the global targeted immunization on a network. Here a total of *n* = 3 nodes are observed. In panel (a), an individual with global information about the network structure chooses the highest degree node *u* to immunize. However, in panel (b), the individual knows only the degree of three nodes in the network at any time, i.e. $v$_1_, $v$_2_, $v$_3_. Consequently, node $v$_3_ with the highest degree *k* = 4 (marked in red) is immunized.

### ER networks

**Figure 2.**
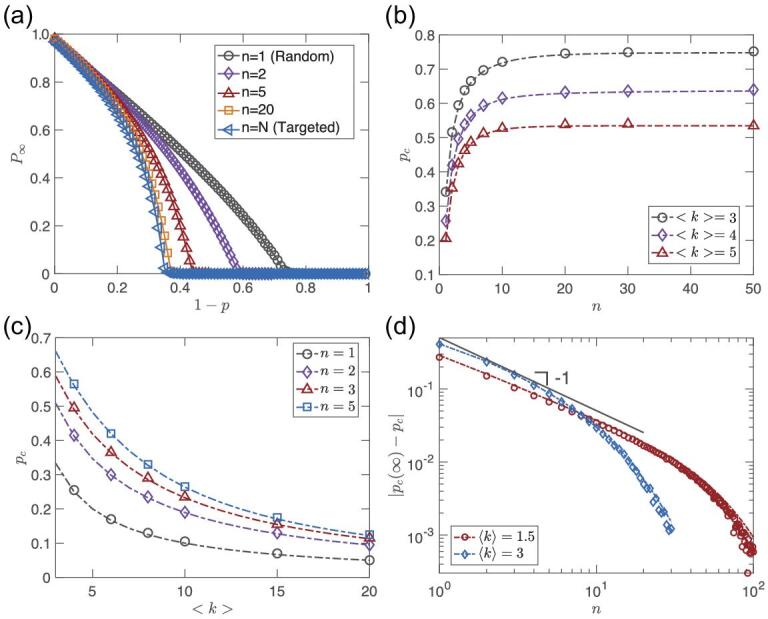
Results on ER networks. (a) The giant component *P*_∞_ of an ER network with 〈*k*〉 = 4 varies with the fraction of immunized nodes 1 − *p* under limited knowledge. As *n* is increased, the limited knowledge immunization tends to have the same immunization effect as targeted immunization. (b) The critical threshold *p*_*c*_ of limited knowledge immunization as a function of *n* on ER networks. The values of *p*_*c*_ were determined from the *p* of the maximal second largest clusters [28]. Note that already for small *n* ∼ 10, *p*_*c*_ is high and close to targeted immunization (global knowledge, *n* ∼ *N*). (c) Critical threshold *p*_*c*_ as a function of the mean degree 〈*k*〉 of ER networks for limited knowledge immunization. (d) The scaling of |*p*_*c*_(∞) − *p*_*c*_| with *n* on ER networks. Symbols are average results of simulations over 100 independent realizations of ER networks with 10^6^ nodes. All simulation results (symbols) agree well with theoretical results of Equation (M10) in the Methods section (dashed lines).

We now study our limited knowledge immunization strategy, i.e. the general result, Equations (M8) and (M9) in the Methods section, on ER networks. First, we analyze the giant component *P*_∞_. For the case *n* = 1, limited knowledge immunization reduces to the classical random immunization, while for *n* → ∞ (meaning that the global network is observed) corresponds to targeted immunization [17,33,35,36]. Using Equation (M9) in the Methods section, the giant component *P*_∞_ can be solved numerically for any given *p*. In Fig. 2(a), simulations and analytical results are shown for the giant component *P*_∞_ as a function of 1 − *p* under limited information immunization for different *n*. As the knowledge index *n* increases from 1 to *N*, the limited knowledge immunization moves from being like random immunization to being like targeted immunization. The simulations are in excellent agreement with the theoretical results (lines).

Next, we focus on the critical threshold, *p*_*c*_, of limited knowledge immunization. Overall, we find that one does not need much knowledge of the network to improve the immunization and *n* ∼ 10 is enough to achieve similar results as for targeted immunization with complete information. This can be seen by observing the critical threshold *p*_*c*_ as a function of *n* in Fig. 2(b). In Fig. 2(c) we show the variation of *p*_*c*_ with 〈*k*〉 for several fixed *n*.

We can also derive the behavior of *p*_*c*_ in the limit of large *n* analytically. By examining Equation (M5) in the Methods section we note that, when *n* → ∞, there are two distinct behaviors depending on whether *k* is small, *F*(*k*) < *p*; or *k* is large, *F*(*k*) > *p*. It can be shown (see Section II.C of the online supplementary material) that the leading term behaves as

(1) \begin{equation*} F_p(k) \sim \left\lbrace \begin{array}{@{}l@{\quad }l@{}} \frac{F(k)}{p}-\frac{1}{n} e^{-\alpha _kn}, & F(k)<p, \\ 1-\frac{1}{n} e^{-\alpha _kn}, & p<F(k)<1, \\ 1, & F(k)=1, \end{array}\right. \end{equation*}

where α_*k*_ = |log[*p*/*F*(*k*)]|. In the limit *n* → ∞, we can get the expected result for targeted immunization, *F*_*p*_(*k*) = min{*F*(*k*)/*p*, 1} [35,36].

Substituting Equation (1) into Equation (M10) in the Methods section and noting that, from a sum of exponentials decaying with *n*, only the lowest decay rate contributes to the leading term, we obtain (see Section II.C of the online supplementary material)

(2) \begin{equation*} p_c(n) \sim p_c^{\infty } - A \frac{1}{n} e^{-\alpha n}, \end{equation*}

where $p_c^{\infty }=p_c(n\rightarrow \infty )$, and the decay rate α is now

$$\begin{equation*}
\alpha =\min _k |\log ( p_c^{\infty } / F(k) ) |.
\end{equation*}$$

The prefactor is $A=(2p_c^{\infty }k_{\rm slow})/(k_> k_<)$, where *k*_<_ is the largest degree such that $F(k)<p_c^{\infty }$, *k*_>_ = *k*_<_ + 1 and *k*_slow_ is the degree that gives the lowest rate α. (See the illustration in the online supplementary material).

It is clear that *k*_slow_ must be *k*_<_ or *k*_>_ because *F*(*k*) is monotonic. If *F*(*k*_slow_) = *F*(*k*_>_) = 1 then *k*_<_ should be taken as *k*_slow_, and the corresponding α should be taken. It should also be noted that if *k*_slow_ is not unique, it would simply change the prefactor *A* in Equation (2). Another special case is where $F(k_{\rm slow})=p_c^{\infty }$; then $|p_c^{\infty }-p_c| \sim 1 /n$ (see Section III of the online supplementary material).

In Fig. 2(d) we show $\Delta p_c=|p_c^{\infty }-p_{c}|$ as a function of *n*. As expected from the theory, we can see that Δ*p*_*c*_ ∼ 1/*n* for small *n* and exponential decay for large *n*. When *p*_*c*_ → 1, which occurs for the ER network when 〈*k*〉 → 1, the power law regime becomes much broader, as explained in Section II.C of the online supplementary material.

### SF networks

**Figure 3.**
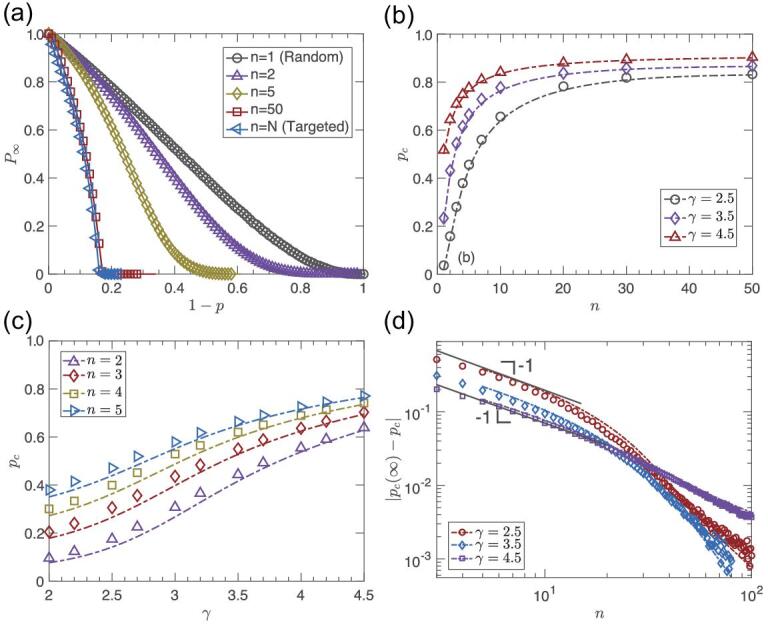
Results for SF networks. Comparison of theory (lines) and simulation (symbols) for limited knowledge immunization, *n*, for SF networks. (a) The size of the giant component versus *n* for the SF network with γ = 2.5. (b) Critical threshold *p*_*c*_ versus *n* on SF networks. (c) The critical threshold *p*_*c*_ as a function of γ for different values of *n*. (d) The log-log plot of |*p*_*c*_(∞) − *p*_*c*_| with *n* on SF networks showing both a power law and exponential behavior. Simulations are obtained for networks of 10^6^ nodes and averages are taken over 100 independent realizations. The minimum and maximum degrees of the network are *m* = 2 and *K* = 1000, respectively. The theoretical results (dashed lines) are calculated from Equation (M10) in the Methods section and are in excellent agreement with the simulation results (symbols).

Next, we study SF networks with *P*(*k*) = *Ak*^−γ^, *k* = *m*, $\cdots$, *K*, where *A* = (γ−1)*m*^γ − 1^ is the normalization factor, and *m* and *K* are the minimum and maximum degrees, respectively [33,34]. Similar to ER networks, the size of the giant component *P*_∞_ can be obtained from Equation (M9) in the Methods section. In Fig. 3(a), we show *P*_∞_ as a function of 1 − *p* for different *n* values. The results demonstrate that SF networks become more immunized as *n* increases. Compared with ER networks, we can observe that slightly higher values of *n* (more knowledge) are needed to reach the near-steady-state region of a fully targeted strategy. In Fig. 3(c) we show how *p*_*c*_ varies with γ for small *n* for the SF network where the minimum and maximum degrees of the network are *m* = 2 and *K* = 1000, respectively. In Fig. 3(d) we show $\Delta p_c=|p_c^{\infty }-p_{c}|$ as a function of *n*. As expected from the theory, Equation (2), the log-log plot captures the slope of −1 for small *n* while an exponential regime exists for large *n*.

For SF networks with 2 < γ < 3, under random immunization (*n* = 1), it has been shown that *p*_*c*_ = 0 for an infinite system [34], while for high-degree immunization (*n* → ∞), *p*_*c*_ > 0 [35,36]. Next we determine for which *n*, *p*_*c*_ becomes nonzero and how it depends on the system size *N*. To this end, we analyze Equations (M5) and (M10) in the Methods section for large *k* (high degrees govern the behavior in SF networks) and small *n* and *p* as follows (elaborated in Section II.D of the online supplementary material). It can be shown that, for large degrees,

$$\begin{equation*}
F(k) \approx 1 - ( k/m )^{1-\gamma }.
\end{equation*}$$

Substituting this into Equation (M5) in the Methods section and assuming that (*k*/*m*)^γ − 1^ ≫ *n* for large degrees, it can be concluded that

(3) \begin{equation*} P_p(k) \approx p^{n-1} P(k). \end{equation*}

In addition, we note that *P*_*p*_(*k*) has a new natural cutoff, *K*_*p*_, which depends on *p* and *N* as (see Section II.D of the online supplementary material)

$$\begin{equation*}
K_{p} \sim p^{n/(\gamma -1)} N^{1/(\gamma -1)}.
\end{equation*}$$

This helps us to evaluate the second moment of *P*_*p*_(*k*):

$$\begin{eqnarray*}
\langle k_{p}^2 \rangle &\sim &\int _{m}^{K_{p}} k^2 p^{n-1} Ak^{-\gamma }\nonumber\\
& \sim & p^{n-1} {K_{p}}^{3-\gamma } \sim p^{n-1+n\beta } N^{\beta }.
\end{eqnarray*}$$

Here β = (3 − γ)/(γ − 1).

Considering this, and substituting Equation (3) into Equation (M10) in the Methods section, keeping the leading terms in the limit of large *N*, we obtain (see Section II.D of the online supplementary material for further details)

(4) \begin{equation*} p_c \sim C(n) N^{-\delta /n} \sim C(n) \exp\! \left[ -\delta \frac{\log N}{n} \right], \end{equation*}

where

$$\begin{equation*}
\delta = \frac{\beta }{1+\beta } = \frac{3-\gamma }{2}
\end{equation*}$$

and

$$\begin{equation*}
\!\!\!\!\!\!\!C(n) = \phi ^{1/n},\quad \phi = \left( \frac{3-\gamma }{\gamma -2} \frac{1}{m} \right)^{(\gamma -1)/2}.
\end{equation*}$$

From Equation (4), it is easy to see that if *n* ≪ log *N* then *p*_*c*_ → 0, while if *n* ∼ log *N* then *p*_*c*_ is nonzero. Note that the prefactor *C*(*n*) depends on *n* but not on *N*.

**Figure 4.**
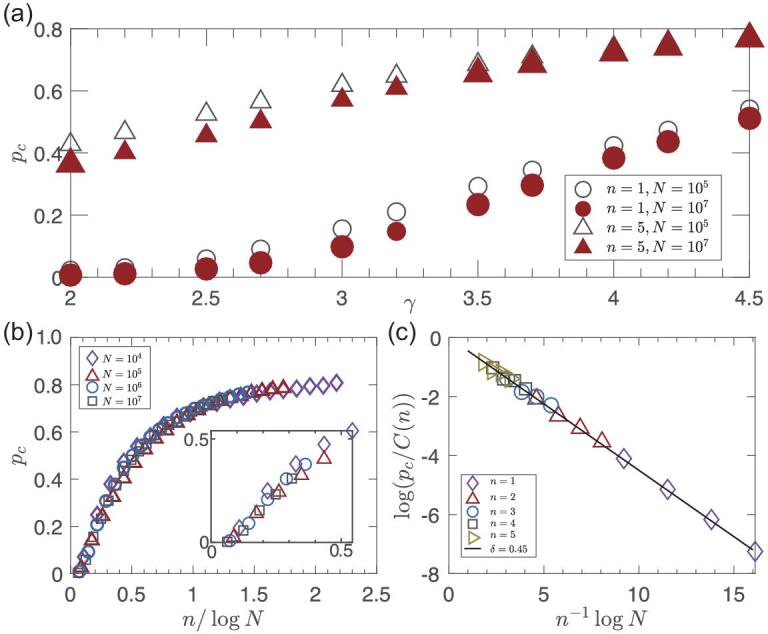
How *p*_*c*_ for SF networks depends on γ and the system size *N*. (a) The critical threshold *p*_*c*_ as a function of γ for different values of *n* and *N*; *p*_*c*_ decreases with increasing *N*. For *n* = 1 and 2 < γ < 3, it is well known that *p*_*c*_ approaches zero for an infinite system. (b) The critical threshold *p*_*c*_ as a function of *n*/log *N* for the SF network with γ = 2.1. The inset shows magnifying the data for small *n* = 1, …, 5; note that *p*_*c*_ approaches zero for *n*/log *N* ≪ 1. (c) The scaling of *p*_*c*_ with *N* and *n* for large *N* and small *n*. Here *C* (*n*) is the prefactor. The minimum and maximum degrees of the nodes are *m* = 2 and *K* = *N*^1/(γ−1)^, respectively. This confirms Equation (4) for γ = 2.1 and δ = (3 − γ)/2 = 0.45.

In Fig. 4(a) we show *p*_*c*_ versus γ. It is known that, for 2 < γ < 3 and *n* = 1, if *N* → ∞ then *p*_*c*_ → 0 [34]. Also, for *n* = 5, we can see that system size matters and *p*_*c*_ decreases as *N* increases. In Fig. 4(b) we show that the scaling with *n*/log *N* of Equation (4) is valid. Furthermore, it can be seen from Fig. 4(b) that, when *n* is small or *N* is large, such that *n*/log *N* ≪ 1 (in Fig. 4(b) it is 0.07), *p*_*c*_ approaches 0. Figure 4(c) supports the exponential scaling of *p*_*c*_ versus *n*^−1^log *N* obtained analytically in Equation (4).

### Real networks

**Figure 5.**
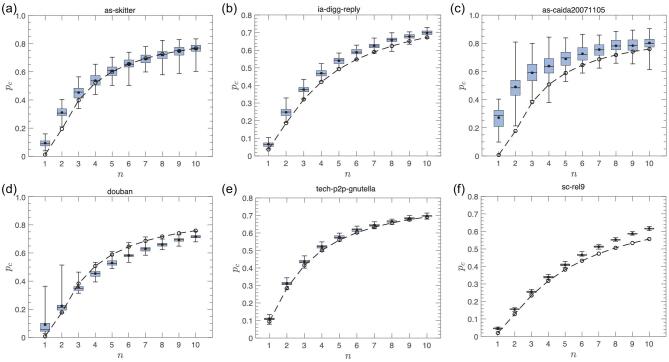
Limited knowledge efficient immunization on six real-word networks (a–f). Each panel is the result of 200 independent simulations. In each panel, filled circles, horizontal lines and dashed lines represent the mean, median value and analytical results of Equation (M10) in the Methods section, respectively. In the simulations, we use the peak of the second largest component to identify the critical point *p*_*c*_.

Our analytical framework is derived and well supported, as shown above, on an ensemble of networks generated by the random configuration model. Here we also test our analytical framework on six real-world networks ranging from computer to social networks. They include (a) autonomous computer networks from the Skitter project [42], (b) the reply network of the social news website Digg [43,44], (c) the network of autonomous systems of the Internet from the CAIDA project [42], (d) the social friendship network from Douban [45], (e) tech-p2p, the eDonkey peer-to-peer network [43,46] and (f) sc-rel9, the scientific computing network [43,47]. Detailed information and statistical features of these networks are summarized in Section IV of the online supplementary material. To test our theory, we execute the limited knowledge immunization strategy on the above real-world networks and use the peak of the second giant component to determine the critical point *p*_*c*_, as shown in Fig. 5. For six real-world networks, we plot the distribution of the critical point over 200 independent realizations, together with the theoretical results. As can be seen from Fig. 5, the critical thresholds *p*_*c*_ for the empirical data are generally consistent with our theoretical predictions. Moreover, we find that our limited knowledge immunization approach is very efficient at preventing outbreaks even for small *n* of order 10.

## DISCUSSION

In summary, our results provide a framework for understanding and carrying out efficient immunization (or efficient attack) with limited knowledge. Especially in cases of global pandemics, e.g. the current COVID-19 epidemic, it is impossible to know the full interactions of all individuals and immunize the most central. We find that an effective way to limit spreading is to obtain information on a few (*n* of the order of 10) individuals and target (test or quarantine) the most central. For example, testers could stand at a supermarket and select a small number of people entering the store simultaneously. Information on the connections of these people, e.g. the number of people they live with or work closely with, where and how often they meet with other people etc., could be obtained quickly through interviews or through cell phone tracking and only the individual with the most connections in the group could be tested, quarantined or immunized. Our results demonstrate that even when this is done in small groups of people (low *n* on the order of 10), it is possible to obtain a significant decrease in global epidemics compared to randomly selecting individuals. In our model, this can be seen by the significantly reduced size of the giant component and the significantly high critical threshold *p*_*c*_. Overall, these findings could help to develop efficient approaches for immunizing large networks and designing resilient infrastructures.

Finally, we note the following two possible variations of our model. In one approach, once we get the degree of a node, we could also obtain partial information (some links) of neighboring nodes and utilize that information to target the highest degree node. We find that adding this knowledge does not change the key results of the proposed model. For detailed results, we refer the reader to Section V of the online supplementary material. Additionally, our immunization approach assumes the existence of a link as binary. Another variant of the model that would be interesting is to incorporate difference frequencies of interactions between different nodes. This would suggest that some nodes are likely to infect some of their neighbors more than others. Such a setting could be implemented through a weighted network, which would be an interesting topic for future study.

## METHODS

Suppose that the degree distribution of a network is given by *P*(*k*), and $F(k)=\sum _{s=0}^{k}P(s)$ is the cumulative probability that the degree of a randomly chosen node is less than or equal to *k*. Furthermore, at an arbitrary time *t* during the iterative percolation process, assume that the distribution of the original degree (including the immunized neighbors) of the remaining nodes is *P*(*k*, *t*). Then the degree distribution of the node that is immunized at time *t* is given by

(M1) \begin{eqnarray*} P_{r}(k,t)&=& F(k,t)^{n}-F(k-1,t)^{n}\nonumber\\ &\equiv &\Delta [F(k,t)^n], \end{eqnarray*}

where *F*(*k*, *t*) is the cumulative distribution of *P*(*k*, *t*). This formula can be recognized as being derived from ‘order statistics’ giving the ‘maximum’ of several independent random variables [48] (see Section II.A of the online supplementary material). For *k* = 0, Equation (M1) becomes *P*_*r*_(0, *t*) = *F*(0, *t*)^*n*^. Hence, we define *F*(*k* = 1, *t*) = 0, and then Equation (M1) is valid for *k* ≥ 0.

In a limited knowledge immunization, each node’s immunization changes the degree distribution of the remaining nodes as

(M2) \begin{equation*} N(k,t+1)=N(k,t)-P_{r}(k,t), \end{equation*}

where *N*(*k*, *t*) is the number of nodes with degree *k* at time *t* and *P*_*r*_(*k*, *t*) is the likelihood that a node immunized at time *t* has degree *k*.

Then, substituting Equation (M1) into Equation (M2) gives

$$\begin{equation*}
N(k,t+1)=N(k,t)-\Delta [F(k,t)^{n}],
\end{equation*}$$

which in the continuous limit becomes

$$\begin{equation*}
\frac{\partial N(k,t)}{\partial t}=-\Delta [F(k,t)^{n}].
\end{equation*}$$

Substituting *N*(*k*, *t*) = (*N* − *t*)*P*(*k*, *t*) yields

$$\begin{eqnarray*}
&&-P(k,t)+(N-t)\frac{\partial P(k,t)}{\partial t}\nonumber\\
&&\quad=-\Delta [F(k,t)^{n}],
\end{eqnarray*}$$

and using *P*(*k*, *t*) = Δ*F*(*k*, *t*), we obtain

$$\begin{eqnarray*}
\Delta \bigg[ -F(k,t)&+&(N-t)\frac{\partial F(k,t)}{\partial t}+ \nonumber\\
&& F(k,t)^{n} \bigg]=0.
\end{eqnarray*}$$

Note that *F*(*k* = −1, *t*) = 0, and thus the entire term inside the Δ is 0 for *k* = −1. Similarly, this implies that, for *k* = 0 and likewise for any *k* ≥ 0, this term is also 0. Thus, we obtain the simple ordinary differential equation

(M3) \begin{equation*} \hspace*{-4pt}(N\!-\!t)\frac{\partial }{\partial t}F(k,t)\!=\!F(k,t)\!-\!F(k,t)^n \end{equation*}

with the initial condition *F*(*k*, *t* = 0) = *F*(*k*). It can be shown (see Section II.B of the online supplementary material) that the solution of Equation (M3) is

(M4) \begin{eqnarray*} \hspace*{-9pt}F(k,t)&=&(1+(F(k)^{1-n}-1)\nonumber\\ &&\times e^{(n-1)\log [(N-t)/N]})^{-{1}/{(n-1)}},\quad \end{eqnarray*}

or, equivalently,

(M5) \begin{equation*} F_p(k)=(1+(F(k)^{1-n}-1)p^{n-1})^{-{1}/{(n-1)}}, \end{equation*}

where *F*_*p*_(*k*) is the cumulative distribution of the degree after immunizing a 1 − *p* fraction of nodes. For *n* = 1, the solution of Equation (M3) is *F*_*p*_(*k*) = *F*(*k*), as expected. Also, Equation (M5) converges to *F*(*k*) in the limit *n* → 1.

We can now obtain the degree distribution of the occupied nodes after a 1 − *p* fraction of nodes are immunized, which is given by

(M6) \begin{equation*} \hspace*{-8pt}P_p(k)\!=\!\Delta F_{p}(k)\!=\!F_{p}(k)\!-\!F_{p}(k\!-\!1). \end{equation*}

The equation for $v$, the probability of a randomly chosen link to lead to a node not in the giant component, is

(M7) \begin{equation*} \hspace*{-6pt}1\!-\!v=\sum _{k=0}^{\infty }\frac{kP(k)}{\langle k \rangle }P({\Theta }|k)(1\!-\!v^{k-1}), \end{equation*}

where *P*(Θ|*k*) is the probability of a node to be occupied given its degree is *k*. This self-consistent equation can be recognized as being derived based on the generating function method [3]. Using Bayes theorem, we note that *P*(*k*)*P*(Θ|*k*) = *P*(Θ)*P*(*k*|Θ) = *pP*_*p*_(*k*). Hence, Equation (M7) becomes

(M8) \begin{equation*} 1-v=\frac{p}{\langle k \rangle }\sum _{k=0}^{\infty }kP_{p}(k)(1-v^{k-1}). \end{equation*}

The giant component is given by

(M9) \begin{eqnarray*} P_{\infty }&=&\sum _{k=0}^{\infty }P(k)P({\Theta } |k)(1-v^{k})\nonumber\\ &=&p\sum _{k=0}^{\infty }P_p(k)(1-v^k), \end{eqnarray*}

where $v$ is found from Equation (M8).

At criticality, we take the derivative of both sides of Equation (M8) and substitute $v$ = 1, representing the location where the first solution with $v$ < 1 exists, as opposed to only the $v$ = 1 solution. Thus, the critical condition is

(M10) \begin{equation*} 1=\frac{p_c}{\langle k \rangle }\sum _{k=0}^{\infty }k(k-1)P_{p_c}(k). \end{equation*}

## Supplementary Material

nwaa229_Supplemental_Files
